# Data on single-step purification method for dye-labeled DNA sequencing

**DOI:** 10.1016/j.dib.2016.02.050

**Published:** 2016-02-27

**Authors:** Kohei Fujikura

**Affiliations:** Kobe University School of Medicine, 7-5-1, Kusunoki-cho, Chuo-ku, Kobe 650-0017, Japan

**Keywords:** DNA sequencing, Purification, Ethanol precipitation, EDTA, Quality value

## Abstract

Dye-labelled DNA sequencing is one of the most common and robust technique required for molecular biology since 1977 (Sanger, 1977) [1]. I have recently provided the single-step purification method for dye-labeled sequencing products, which is based on the removal of the washing step in EDTA/ethanol precipitation (Fujikura, 2015) [2]. Here I assess and report the accumulated data of the modified method on the larger scale in practice.

**Specifications Table**

**Table:** 

Subject area	*Molecular biology*
More specific subject area	*DNA sequencing*
Type of data	*Graph*
How data was acquired	*DNA sequencer*
Data format	*Analyzed data*
Experimental factors	*Dye-labeled sequencing reaction is performed in specified dilution rate.*
Experimental features	*The modified EDTA/ethanol method skips the washing step in EDTA/ethanol precipitation for dye-labeled DNA sequencing.*
Data source location	*Japan*
Data accessibility	*The data are with this article.*

**Value of the data**

• The data provide the scheme and characterize the success rate for modified EDTA/ethanol purification method for dye-labeled DNA sequencing.
• The data provide the optimization of methods, including the amount of DNA template, primers, and buffer conditions for rapid purification of dye-labeled sequencing.
• The modified method provides the quick and inexpensive purification technique for DNA sequencing.

## 1 Data

**Fig. 1:**
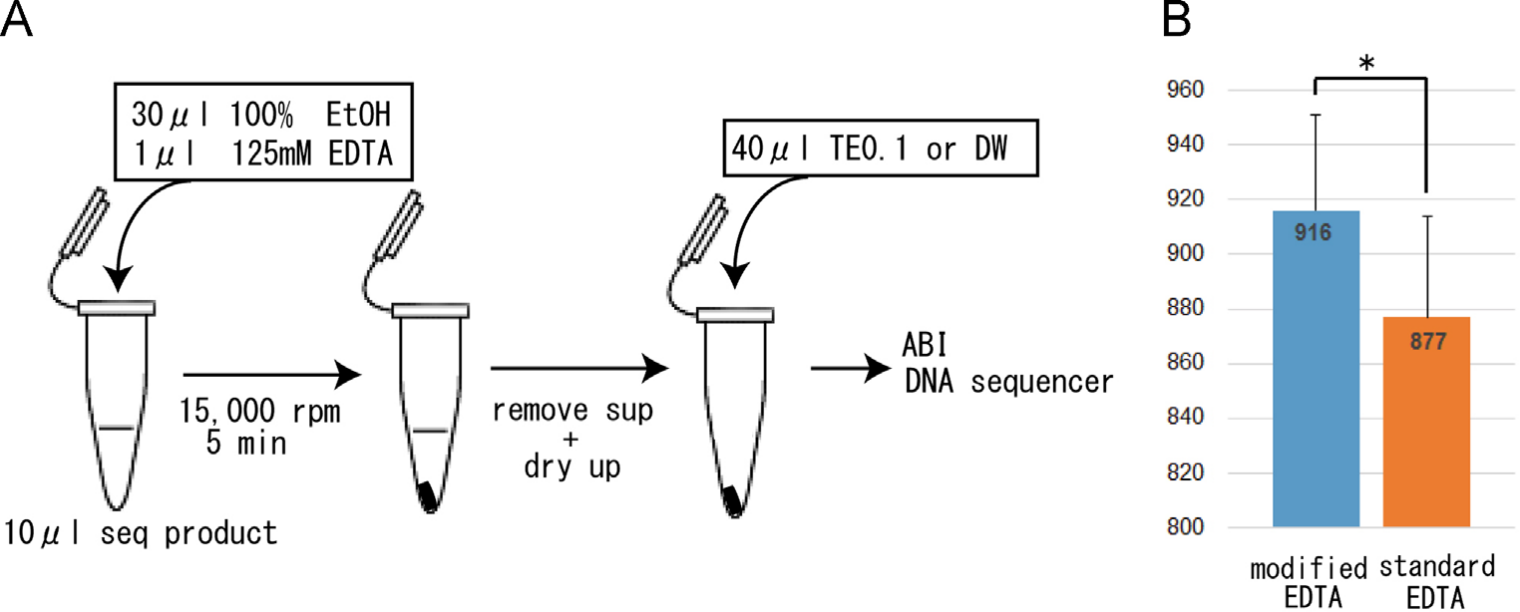
(A) Method for the single-step purification of dye-labeled sequencing products is described in left half. (B) Comparison of the sequence read lengths with QV>20 in two different methods is shown in right graph. Results are shown as the means±standard deviations (Student *t* tests; ^∗^*P*<0.01).

Dye-labeled DNA sequencing technique is still an important tool for clinical decision making on cancer [3], [4], [5], drug metabolism genotyping [6], [7], [8], pathogen identification [9], [10], [11], inherited disease [12], [13], [14], [15], [16], [17] and so on [18], [19]. However, the methods remain laborious and time-consuming. Here I modified sequencing method and assessed its data quality (Fig. 1). The modified method requires only 10 min, whereas commercial purification kits and standard ethanol precipitation require a longer processing time. DNA sequences of more than 850–900 bp were more stably obtained in a single sequencing reaction with this rapid method without compromising high-quality base calling and read length (916±35 bp, *n*=168, QV>20). (Fig. 1). The read length obtained by this method was significantly longer than that obtained by standard methods (modified EDTA vs. standard EDTA (877±37 bp, *n*=168); *P*<0.01) (Fig. 1). The failure rate of modified method was quite low (0.6%; 1/168).

## 2 Experimental design, materials and methods

### 2.1 Sequencing reaction

Sequencing reaction was performed in a 10 μl scale using the BigDye Terminator v3.1 Cycle Sequencing Kit (dilution rate: 1:16–1:32) (Applied Biosystems, Foster City, CA, USA), 10 pmol of various primers, and 50–1500 ng of template DNA (50 ng for 100–300 bp PCR product, 250 ng for 300–2000 bp PCR product, or 1500 ng for plasmid) for one experimental run. The following thermal cycle was used for the amplifications: 96 °C for 1 min, followed by 40–50 cycles of 96 °C for 10 s, 50–58 °C for 5 s and 60 °C for 150 s.

### 2.2 Modified purification technique and data analysis

1 μl of 125 mM of EDTA and 30 μl of 100% ethanol were added to each sequencing reaction, and the mixture was then lightly vortexed (Fig. 1). After centrifugation (15,000*g*, 5 min for tube; 4000 g, 15 min for plate), the supernatant was carefully aspirated and discarded. Next, after drying the precipitate, 40 μl of distilled water or TE0.1 buffer (10 mM Tris–acetate [pH 8.0] + 0.1 mM EDTA) was added to each reaction. ABI PRISM 3130 Genetic analyzer and POP-7 polymer were used as the separation machine and matrices, but the other Genetic analyzer (ABI Prism 310, 3730, or 3500) and polymers (POP-4 or POP-6) are also applicable. The sequence data was analyzed with ABI Prism DNA Sequencing Analysis Software v5.1. The quality value score 20 (QV20) was used as indicator of sequencing quality. Student׳s *t* tests were performed to evaluate the significance between two methods.
